# Imidacloprid Resistance Challenges in Brazilian Strains of *Drosophila suzukii* (Diptera: Drosophilidae)

**DOI:** 10.3390/insects16050494

**Published:** 2025-05-05

**Authors:** Felipe Andreazza, Flávio Roberto Mello Garcia, Pedro Bento da Silva, Lucas Bretas Barbosa, Joel Marques de Oliveira, Gabriel Netto Araújo, Eugenio E. Oliveira

**Affiliations:** 1 Departamento de Entomologia, Universidade Federal de Viçosa (UFV), Viçosa 36570-900, MG, Brazil; 2 Department of Ecology, Zoology and Genetics, Biology Institute, Federal University of Pelotas, Pelotas 96010-900, RS, Brazil

**Keywords:** spotted wing *Drosophila*, insecticide toxicity, neonicotinoid resistance, cytochrome P450, resistance management

## Abstract

*Drosophila suzukii* (Matsumura) is a relevant pest affecting berries and stone fruits globally, including in the Neotropical region, where its invasion was reported about a decade ago. Despite chemical control being the main management method for *D. suzukii*, data on insecticide susceptibility in Neotropical *D. suzukii* populations are scarce. Here, we assessed the susceptibility of three field-collected Brazilian *D. suzukii* populations to four insecticides (i.e., deltamethrin, permethrin, spinetoram, imidacloprid) and contrasted this with a standard insecticide-susceptible population. Using the discriminating concentration (LC_90_) from the standard susceptible population, we identified resistant populations. Synergist exposure (piperonyl butoxide, triphenyl phosphate, diethyl maleate) indicated the role of detoxification enzymes in resistance. Our results showed that deltamethrin was the most toxic, followed by spinetoram, permethrin, and imidacloprid. While all field populations were similarly susceptible to pyrethroids and spinosyns, one population from Minas Gerais (i.e., *Paula Candido*) had significantly lower imidacloprid susceptibility, with only 53.4 ± 5.2% mortality at 10.0 g/L (the equivalent of 10-fold the estimated imidacloprid LC_90_). Only piperonyl butoxide increased the imidacloprid susceptibility of *Paula Candido* flies. Our findings indicate the occurrence of cytochrome P450 enzyme-based imidacloprid resistance in the state of Minas Gerais, which can challenge the management of *D. suzukii* in Brazil.

## Simple Summary

This study investigates the susceptibility of field-collected Brazilian *Drosophila suzukii* populations to four insecticides: deltamethrin, permethrin, spinetoram, and imidacloprid. Our findings reveal relevant resistance to imidacloprid in a population from Minas Gerais (i.e., *Paula Candido*), a key region for strawberry production in Brazil. This resistance is linked to the activity of detoxification enzymes, particularly cytochrome P450, which play a crucial role in the pest’s ability to survive insecticide exposure. With limited information on insecticide susceptibility in Neotropical *D. suzukii* populations, this study highlights the urgent need for effective pest management strategies that consider the evolving resistance patterns. Overall, the results indicate that addressing imidacloprid resistance is essential for the sustainable management of *D. suzukii* in Brazil, where the economic impact on fruit production is relevant. This research contributes valuable insights into the dynamics of pest resistance and the implications for agricultural practices in the region.

## 1. Introduction

*Drosophila suzukii* possesses biological characteristics such as a short generation time, high reproductive outputs, and behavioral and physiological plasticity [1,2,3]. These traits, combined with their very high polyphagia [4], make its management a complex task [5]. Notably, the economic impact of this invasive species is substantial, with *D. suzukii* now distributed across more than 296 municipalities in Latin America and affecting over 60 host plants in 25 families [5]. For instance, in South America, *D. suzukii* causes significant losses to horticulture, with estimated direct losses per year ranging from 1.2 to 2.7 tons/ha of cherries, translating to about USD 5000 to USD 17,550/ha [6]. Furthermore, losses have been estimated at 1.0 to 1.5 tons/ha, equivalent to USD 4000/ha in Chilean blueberry orchards [6]. In Brazil, the average economic losses for peaches reach USD 21.4 million annually, while figs incur losses of USD 7.8 million [7]. In Argentina, although not quantified economically, larvae in cherries pose challenges for exporting these fruits to Australia and New Zealand due to quarantine restrictions [8].

Despite the proposal of various control strategies [5,9,10,11], farmers heavily rely on insecticide sprays in their fields [12,13]. However, the need to use a few groups of molecules, combined with the inappropriate use of these control tools, has been shown to contribute to the selection of resistant populations of *D. suzukii* under controlled conditions [14,15,16]. Recent reports have identified other undesirable effects of this small number of available molecules, which include the identification of a field population of *D. suzukii* resistant to spinosad [17], stimulatory reproduction on sublethally exposed *D. suzukii* [18], and detrimental effects to *D. suzukii* parasitoids [19,20].

There is, therefore, an increasing need for additional studies focusing on insecticide resistance monitoring [17,21,22,23,24] due to the potential damages caused by these organisms in the production of berries and stone fruits worldwide, including in the Neotropical region. In this scenario, the Brazilian production of soft-skinned fruits has been damaged by the activities of drosophilids, including *D. suzukii* [5,25,26]. *Drosophila suzukii* has been present since 2013 [27] and has spread to several geographical regions and hosts [5,25,26,28,29]. Several insecticides, especially pyrethroids and neonicotinoids, are frequently used in the management of *D. suzukii* or other pests that attack soft-skinned fruits in Brazil [5,9,24,26,29], but only insecticides from the spinosyn chemical group are registered for controlling *D. suzukii* in Brazil [30]. Interestingly, recent research using Brazilian populations of *D. suzukii* could not relate any potential insecticide control failures to the selection of resistant individuals [24]. However, these findings did not include populations from strawberry-producing regions of the Minas Gerais state, where *D. suzukii* was initially reported in 2016. Such strawberry-producing regions in Minas Gerais also cultivate other predominant crops, e.g., coffee (*Coffea arabica* L.) and guava (*Psidium guajava* L.), in which the number of neonicotinoid applications occur multiple times throughout the growing season, either alone or in combination with fungicides [31,32]. This practice may inadvertently select for insecticide-resistant flies, as *D. suzukii* may survive in overripe, decaying coffee bean pulps, a phenomenon previously observed in other fly species [33] during periods of scarcity of preferred hosts. In guava orchards, for instance, farmers are heavily dependent on the application of neonicotinoids for controlling insect pests (e.g., psyllids), and are compelled to spray guava trees at intervals of 15 to 20 days to protect the new shoots [34,35].

Here, we first assessed the susceptibility to three groups of insecticides on a population collected at the onset of the South American invasion which were kept in an insecticide-free environment since then. This population is here termed *Pelotas* and is considered our standard susceptible population. We contrasted the susceptibility of *Pelotas* individuals with the susceptibility recorded from individuals of three field-collected populations. By using synergists, we further assessed the potential of detoxification mechanisms as underlying the likely resistance to these insecticides. Our findings are expected to assist in the development of management strategies and global resistance monitoring efforts.

## 2. Materials and Methods

### 2.1. Insect Rearing and Chemicals

**Figure 1:**
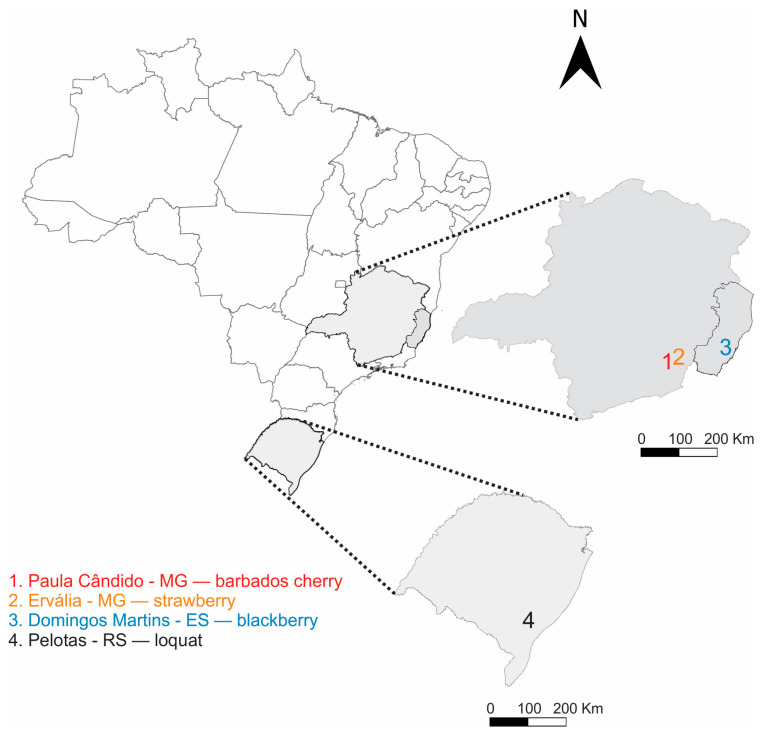
Sampling sites and hosts (crop types) where the original individuals of *Drosophila suzukii* were collected.

The bioassays and fly rearing were conducted in laboratory conditions of 25 ± 2 °C, an RH of 50 ± 10%, and a 12 h photophase. The strains were started with individuals collected as larvae in infested fruits from the field at different sites and dates (Figure 1). The flies were then reared in the laboratory on an artificial diet following the methodology previously described elsewhere [1]. The reference strain in the susceptibility bioassays was originally collected from loquat fields in September of 2014, in a region recently invaded (approximately 2 years after first detection [27]), located in the county of *Pelotas* (Rio Grande do Sul state, Brazil). Two other populations were established from infested, field-collected fruits in the state of Minas Gerais (Brazil). The first was obtained in March 2016 from an organic strawberry field in *Ervália* county [36] and the second was collected from a rural house orchard with mixed fruit species in *Paula Cândido* county in January 2018. The fourth strain was originally collected from strawberry and blackberry orchards in *Domingos Martins* county (Espírito Santo state, Brazil) in the years of 2016 and 2017. Except for the Pelotas strain, which was already in its 48th generation under controlled conditions, the other strains had their insecticide susceptibility assessed as soon as they had enough individuals. Six generations were required for the Domingos Martins and Ervália strains, and eight generations for the Paula Cândido strain to obtain approximately 1000 adult flies of the same age (i.e., 3–4 days) per generation, enabling the assessment of susceptibility to the insecticides. The generations conducted under laboratory conditions were reared in an insecticide-free environment.

The synthetic insecticides used in this study were obtained from the local market and were formulated as commercial insecticides. We used the pyrethroids deltamethrin (type II pyrethroid, water-dispersible granules at 25 g active ingredient (a.i.)/L, Bayer CropScience, São Paulo, SP, Brazil) and permethrin (type I pyrethroid, 250 g a.i./L, emulsifiable concentrate, BASF Corporation, Raleigh, NC, USA), the neonicotinoid imidacloprid (water-dispersible granules at 700 a.i./L; Bayer CropScience, São Paulo, SP, Brazil) and the spinosyn spinetoram (water-dispersible granules at 250 g a.i./L; Corteva Agriscience, Indianapolis, IN, USA).

### 2.2. Toxicity Exposure Protocol

For all the toxicity bioassays, the exposure procedures followed the IRAC protocol No: 02631 [37], which is recommended for bioassays with *Musca domestica* L. (Diptera: Muscidae) adults, with slight modifications made for use with *D. suzukii* [9]. Each sample unit consisted of a 200 mL glass jar containing a 2 cm long dental cotton wick impregnated with 1.9 mL of the chemical solution at the desired concentration. The top of each jar was sealed with a foam plug. Inside the jar, we released 20 to 25 flies, aged 3 to 4 days, that were randomly collected from the rearing cage with a hand aspirator. After 24 h, the flies’ mortality was visually assessed. Flies that did not show movement even after stimulation with a fine brush were considered dead. All the compounds were diluted in a pre-made solution of water with 20% sugar (*m*/*v*), which also consisted of the solution used as a control treatment.

### 2.3. Insecticide Susceptibility and Resistance Bioassays

Concentration–mortality response curves were determined using the standard susceptible population. For that, pre-tests were performed using four replicates for each of the 4 to 5 logarithmically spaced concentrations of the selected compound to determine the concentration range in which each compound caused mortality of the exposed flies between 0 and 100%. The exposure bioassays were repeated using six to seven concentrations within the determined ranges, with four replicates per concentration, and the mortality data were then used to estimate the lethal concentrations (LCs) for each compound. To assess the potential resistance, we used different *D. suzukii* strains originally collected in southeastern Brazil and compared them to our standard susceptible population (Figure 1). Adult flies from these different strains were exposed to a discriminatory concentration of each insecticide, estimated to kill 90% (LC_90_) of the exposed individuals of our standard susceptible population. Four replicates were used for each population/insecticide combination as well as for the control treatment within each population. The mortalities were assessed at 24 h, following the same protocol mentioned earlier.

### 2.4. Bioassays with Synergized Imidacloprid in Paula Candido Population

To confirm the occurrence of resistance and investigate its potential underlying mechanisms (i.e., metabolic vs. target site modification), we exposed *Paula Candido* individuals to 5000 mg/L and 10,000 mg/L of imidacloprid, which corresponded to five- and 10-fold the LC_90_ estimated for individuals of the *Pelotas* population. The *Paula Candido* individuals were either pre-exposed (1 h of exposure) or not exposed to synergists involved in the inhibition of detoxification enzymes. We used piperonyl butoxide (an inhibitor of cytochrome P450-dependent monooxygenases and esterases), diethyl maleate (an inhibitor of glutathione S-transferase), and triphenyl phosphate (an esterase inhibitor). We then compared the mortality caused by the insecticide alone and that synergized by these enzyme inhibitors. For this bioassay, 2 mL of acetone solution containing one of the three synergists was applied inside each vial prior to the insecticide exposure. The vials were rolled over on the bench top until the solution had dried, leaving the inner walls of the glass vials coated with the enzyme inhibitors. The synergist concentrations used to coat the vial walls were determined in pre-tests, as a higher concentration did not cause any fly mortality. These concentrations were 100 mg/L for piperonyl butoxide and 500 mg/L for triphenyl phosphate and diethyl maleate. The adult flies were released inside the vials shortly after the enzyme inhibitor solution that was applied had dried. After one hour of the flies being in contact with the synergists, an imidacloprid-impregnated cotton wick was placed inside the vials, allowing a further 24 h period of insecticide and synergist exposure. At the end of the bioassay, the mortality was evaluated following the same procedures already described.

### 2.5. Data Analysis

The mortality data of the concentration–response bioassays were submitted to a Probit analysis [PROC PROBIT [38]]. The toxicity ratios among the used insecticides were calculated according to Robertson et al. (2007) [39]. The data from the resistance screening bioassay were submitted to a Z-test for a binomial proportion with continuity adjustment [40] (PROC POWER [38]). Finally, the mortality of each of the synergist treatments was compared with its insecticide treatment control by Dunnett’s test within One Way ANOVA performed on Sigma Plot 12.5 (Systat Software, San Jose, CA, USA) (*p* < 0.05) after checking the assumptions of the normality of the residues and the homogeneity of variances.

## 3. Results

### 3.1. Concentration–Response Bioassay Data

**Figure 2:**
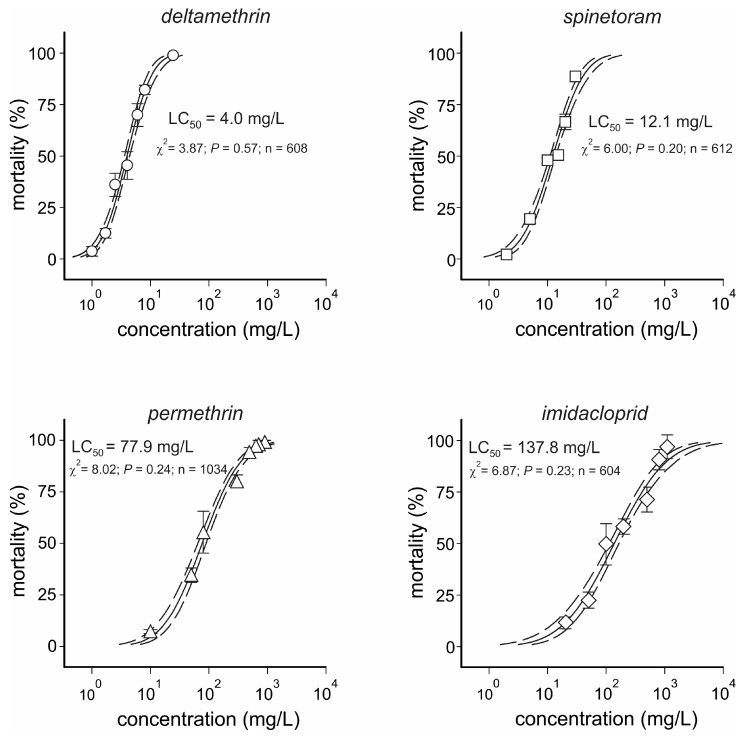
Concentration mortality curves of different synthetic insecticides on *Drosophila suzukii* adults of the standard susceptible population (*Pelotas*). The symbols represent the observed mean mortality (±SEM), while the continuous lines represent the Probit-predicted mortality responses for each insecticide compound. Dashed lines represent the 95% upper and lower confidence limits for the estimated concentrations. LC_50_ = lethal concentration expected to kill 50% of exposed individuals. Please refer to Table S1 for statistics.

**Figure 3:**
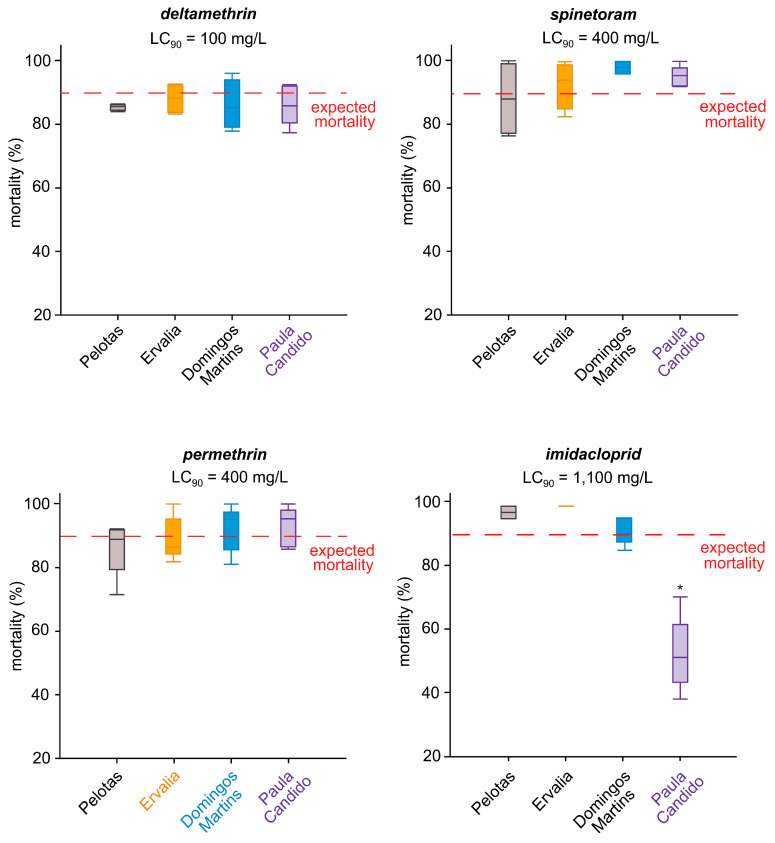
Mortality (±SEM) of *Drosophila suzukii* individuals of four populations (*Pelotas*, *Ervalia*, *Domingos Martins*, and *Paula Candido*) exposed to a diagnostic concentration (i.e., LC_90_) of insecticides. The horizontal dashed line represents the expected mortality (i.e., 90%). Asterisks indicate a significantly (*p* < 0.05) lower mean observed mortality than the Z-test limit for the expected mortality.

The concentration–response bioassay data for the standard susceptible population and all the insecticides tested were fitted in the Probit analysis with low χ^2^ values (Supplementary Table S1, Figure 2). Deltamethrin was the most toxic insecticide (LC_50_ = 4.0 mg/L), followed by spinetoram (LC_50_ = 12.1 mg/L with a toxicity ratio (TR_50_) of 3.1), permethrin (LC_50_ = 77.9 mg/L and a TR_50_ of 19.3), and imidacloprid (LC_50_ = 137.8 mg/L and a TR_50_ of 34.7). Using the discriminatory concentrations (LC_90_) of each tested insecticide, our toxicity results revealed only one resistance case, where imidacloprid LC_90_ (1100 mg/L) caused 53.4 ± 5.2% mortality for individuals of the *Paula Candido* population (Figure 3), which was significantly different from the expected mortality (Z-test, *p* < 0.05). All the other tested combinations had an observed mortality higher than the lower Z-test limits (*p* < 0.05) and were therefore considered susceptible populations.

### 3.2. Synergism Bioassay

**Figure 4:**
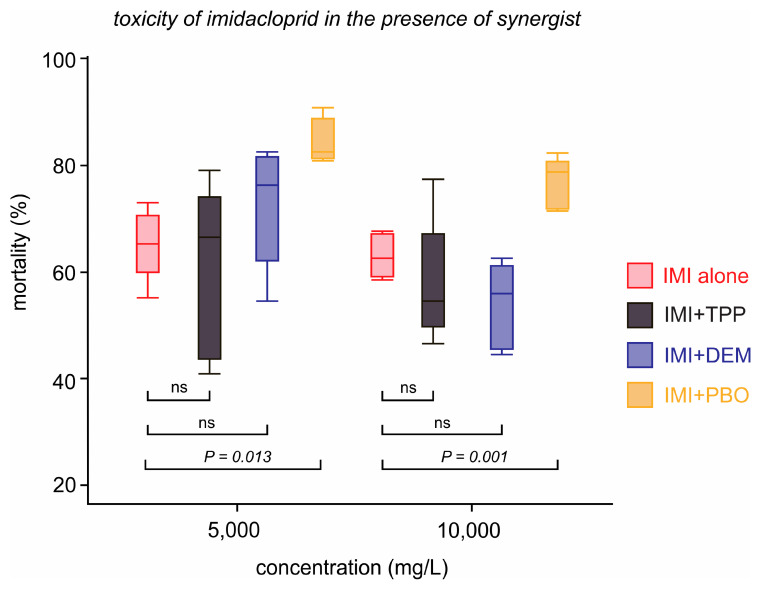
Effects of the synergists piperonyl butoxide (PBO), diethyl maleate (DEM), and triphenyl phosphate (TPP) on the mortality of *Drosophila suzukii* individuals of the *Paula Candido* population caused by imidacloprid at 5.0 g/L (five-fold the estimated LC_90_ on the standard susceptible population) and 10.0 g/L (10-fold the estimated LC_90_ on the standard susceptible population). “ns” indicates no significant differences (paired *t*-test; *p* < 0.05) between the synergized and un-synergized imidacloprid toxicities.

All imidacloprid- and synergist-unexposed flies survived in the synergism bioassay. When exposed to imidacloprid concentrations as high as 5.0 g/L and 10.0 g/L alone, the mortality levels were never higher than 70%, further confirming their resistance to this insecticide (Figure 4). Among the three synergists used, only piperonyl butoxide was capable of significantly increasing the mortality when compared to imidacloprid alone in both 5000 mg/L (*F*_(3,16)_ = 4.90; *p* = 0.013) and 10,000 mg/L (*F*_(3,16)_ = 8.33; *p* = 0.001) imidacloprid concentrations (Figure 4).

## 4. Discussion

Here, we reported a case of insecticide resistance in a Neotropical strain of *D. suzukii* for the first time. We demonstrated that adult individuals of the *Paula Candido* strain could resist 10,000 mg/L of imidacloprid, equivalent to a 10-fold increase compared to the estimated imidacloprid LC_90_ in our standard susceptible population (*Pelotas*). Furthermore, by using the different synergists, we could demonstrate that piperonyl butoxide (PBO) was the only molecule capable of increasing the imidacloprid-mediated toxicity in *Paula Candido* individuals, which indicated the potential involvement of cytochrome P450 enzymes in the imidacloprid resistance.

Neonicotinoids are agonists that mimic the action of acetylcholine, acting on different nicotinic acetylcholine receptor (nAChR) subtypes [41,42] by desensitizing these transmembrane proteins, making these receptors less responsive to acetylcholine, disrupting the nervous system information transmission, and eventually killing the insects [42,43,44]. Mutations in the target sites (i.e., nAChR subtypes) have been identified as the primary cause of neonicotinoid resistance in several pests [45,46,47]. However, as demonstrated in our study, the imidacloprid resistance has also been linked to the over-expression of detoxification enzyme genes, particularly the CYP family, which encodes cytochrome P450 enzymes [45,46,47].

Cases of reduced insecticide susceptibility in *D. suzukii* are still scarce, but some have been reported in the USA for pyrethroid, organophosphate, and spinosyn insecticides [17,21,22]. For the spinosyn spinosad, *D. suzukii* flies from a field site were largely less susceptible and were considered insecticide-resistant [17], while for the other insecticides, differences in susceptibility were still small [21,22]. Interestingly, at least for malathion and under laboratory selection pressure, the resistance did not evolve [48], suggesting either a lack of resistant alleles in that population or strong fitness costs associated with it. Correlations between reported cases and potential new reports of resistance are typically established only after the genetic characterization of the involved resistance allele(s). This requirement has not been met by any previous studies or the current report. The molecular characterization of resistance will enhance the understanding of the origins of allele(s) of resistance, relating them to those reported in individuals from other invaded regions [28]. However, our primary focus here was to predict the potential susceptibility issues of Brazilian strains of *D. suzukii* to commercial formulations and identify preliminary resistance mechanisms by using synergists to map the involvement of metabolic enzymes.

Despite the unknown origin of the resistance allele involved in the observed resistance, its frequencies seem to have quickly increased in a restricted area since the *Ervalia* population, collected only 24 km from the *Paula Candido* site, was susceptible to all of the insecticides tested. The rapid selection of insecticide resistance can result from the pest biology, management strategies, and the naturally occurring high number/frequency of resistance sources, which are not uncommon [49,50]. *Drosophila suzukii* from two field locations in the USA was recently demonstrated to differentially express a complex high number of detoxification genes as well as have variable numbers of single nucleotide variant mutations, indicating a potentially high resistance source in this species [22]. A high number of resistance sources, from target site mutations [51,52,53] to detoxification [54,55], also occurs in other drosophilid species studied worldwide.

Our findings, in combination with previous results (e.g., [17,24]), certainly do not provide a full panorama of the insecticide resistance sources and their field frequencies in this species in Brazil. It is noteworthy that for the six generations required to conduct our discriminatory dose bioassay in the Ervalia and Domingos Martins strains, the flies were maintained in an insecticide-free environment. This condition can result in a rapid decline in resistance allele frequencies to below detectable levels. However, the Paula Candido strain still presented resistance even after eight generations in this insecticide-free environment. Surveys covering larger areas and continuous insecticide resistance monitoring studies using rapid/cheap toxicological assessment methods are needed [56]. This should mitigate the risks of resistance selection and control failure in this species. Moreover, despite lacking selectivity to natural enemies [19,20], a wide range of insecticides continue to offer the effective control of this pest on a global scale [21], and together with novel and recently synthesized molecules which are less harmful to natural enemies [57], they remain a viable option for rotation.

Currently (i.e., as far as December 2024), there is only one insecticide (a spinosyn) registered with government controlling agencies for use against *D. suzukii* in Brazil. However, neonicotinoid insecticides such as imidacloprid, acetamiprid, and thiamethoxam are allowed for application on some of *D. suzukii*’s main hosts, such as strawberries, guavas, peaches, and grapes [30]. In the sampled region, coffee (*Coffea arabica* L.) is the predominant crop, and it is treated with high doses of neonicotinoids, either alone or in combination with fungicides, to control coffee pests [58,59]. While it remains unclear whether *D. suzukii* could be exposed to these neonicotinoid products, during periods of preferred host scarcity, the species may survive by feeding on overripe decaying coffee bean pulp, similar to other fruit flies like *Anastrepha fraterculus* (Wiedemann) and *Ceratitis capitata* (Wiedemann) [60]. It is important to note that worldwide, although neonicotinoids are less effective than pyrethroids, organophosphates, and spinosyns in managing *D. suzukii* infestations [61,62], they are still used in rotation. The potential for neonicotinoids to select resistant individuals, combined with their lower efficacy compared to other options, their potential stimulatory reproduction in sublethally exposed individuals, and their negative impacts on non-target species [18,63], raises concerns about their continued use against *D. suzukii*.

Among the insecticides tested in this study, the pyrethroid deltamethrin and the spinosyn spinetoram shared the stage as the most toxic ones and, therefore, could be effectively used in rotation with other products to break any possible selection cycle to imidacloprid. The same is true for permethrin, since both populations originally collected from Minas Gerais state did not present differences in susceptibility to this insecticide. Nevertheless, spinetoram is being used more frequently than recommended to control *D. suzukii* in Latin America, which may result in the selection of resistant individuals [5]. Malathion, representing an organophosphate, did not successfully select a resistant population in laboratory selection bioassays [48] and is also effective against *D. suzukii* populations worldwide [14,21,64], and thus should also be considered for rotation with other chemical classes.

Thus, by demonstrating a differential susceptibility to imidacloprid in a *D. suzukii* population from Minas Gerais state, our findings indicate a potential source of neonicotinoid resistance in Brazilian populations of these invasive flies. Further field surveys are surely necessary to avoid future control failures if a neonicotinoid is ever registered to be used against *D. suzukii* in Brazil. Since no information on potential neonicotinoid resistance in *D. suzukii* from other invaded regions (e.g., North America, Africa, and Europe) has yet been reported, the current worldwide recommendation for area-wide integrated pest management [12,65,66] with special attention to the rotation of efficient insecticides covering different modes of action [67] is naturally recommended.

## Data Availability

The data that support the findings of this study are available from the first and corresponding authors upon reasonable request.

## Supplementary Materials

The following supporting information can be downloaded at: https://www.mdpi.com/article/10.3390/insects16050494/s1, Table S1: Toxicities and toxicity ratios of different insecticides in the *Drosophila suzukii* standard susceptible population (*Pelotas*); Figure S1: Illustrative representations of the cages (**A**), diets (**B**), and experimental units (**C**) used for rearing and conducting insecticide toxicological biological assays against *Drosophila suzukii*.
