# Supplementary material for: Imidacloprid Resistance Challenges in Brazilian Strains of Drosophila suzukii (Diptera: Drosophilidae)
Source: Insects. 2025 May 5;16(5):494. doi: 10.3390/insects16050494 (PMC12112340; doi:10.3390/insects16050494)
Supplement: Supplementary file 1 [file insects-16-00494-s001.zip › insects-3584591-supplementary.pdf]

Supplementary Materials

Supplementary Table S1. Toxicities and toxicity ratios of different insecticides in the *Drosophila suzukii* standard susceptible population (*Pelotas*).

Insecticide	LC_{50} (CI) – g/L	*n*	χ^2	*d.f*	*P*	TR_{50} (CI)
Deltamethrin	0.004 (0.003 – 0.005)	608	3.87	5	0.57	1.0 (0.9 – 1.1)
Spinetoram	0.012 (0.011 – 0.014)	612	6.00	4	0.20	3.1 (2.8 – 3.4)
Permethrin	0.077 (0.065 – 0.089)	1034	8.02	6	0.24	19.3 (17.1 – 21.8)
Imidacloprid	0.138 (0.113 – 0.166)	604	6.87	5	0.23	34.7 (24.2 – 49.6)

LC_{50} = lethal concentration; n = total number of insects used; TR_{50} = toxicity ratio; CI = confidence interval.

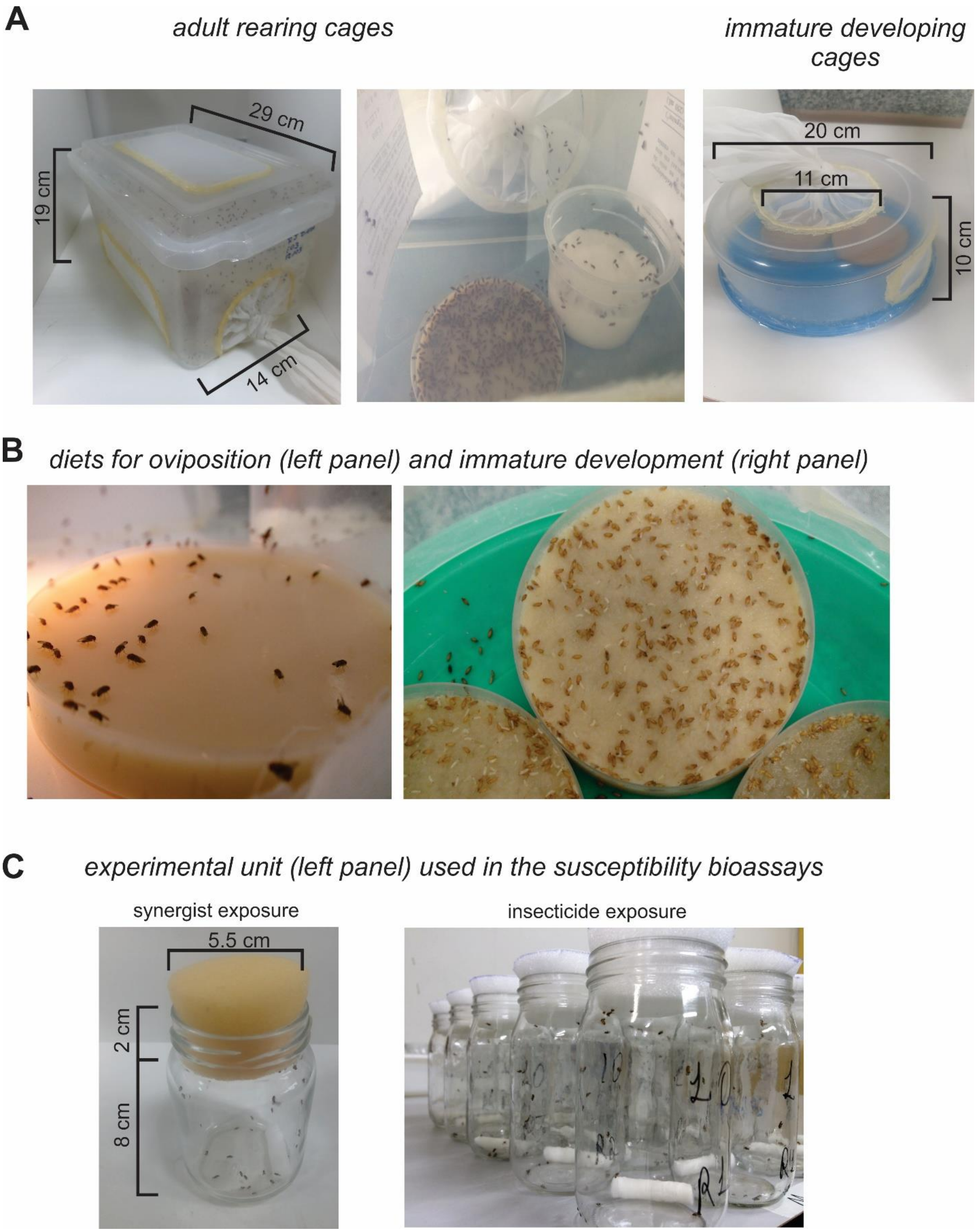

Supplementary Figure S1. Illustrative representations of the cages (***A***), diets (***B***) and experimental units (***C***) used for rearing and conducting insecticide toxicological biological assays against *Drosophila suzukii*.
